# The International Conference on Intelligent Biology and Medicine (ICIBM) 2016: summary and innovation in genomics

**DOI:** 10.1186/s12864-017-4018-6

**Published:** 2017-10-03

**Authors:** Zhongming Zhao, Zhandong Liu, Ken Chen, Yan Guo, Genevera I. Allen, Jiajie Zhang, W. Jim Zheng, Jianhua Ruan

**Affiliations:** 1 Center for Precision Health, School of Biomedical Informatics, The University of Texas Health Science Center at Houston, Houston, TX 77030 USA; 2 Human Genetics Center, School of Public Health, The University of Texas Health Science Center at Houston, Houston, TX 77030 USA; 3 The Jan and Dan Duncan Neurological Research Institute, Baylor College of Medicine, Houston, Texas 77030 USA; 4 Department of Bioinformatics and Computational Biology, The University of Texas MD Anderson Cancer Center, Houston, TX 77030 USA; 5 Departments of Cancer Biology and Biomedical Informatics, Vanderbilt University Medical Center, Nashville, TN 37232 USA; 6 Department of Statistics, Rice University, Houston, TX 77005 USA; 7 School of Biomedical Informatics, The University of Texas Health Science Center, Houston, TX 77030 USA; 8 Department of Computer Science, The University of Texas at San Antonio, San Antonio, TX 78249 USA

## Abstract

In this editorial, we first summarize the 2016 International Conference on Intelligent Biology and Medicine (ICIBM 2016) that was held on December 8–10, 2016 in Houston, Texas, USA, and then briefly introduce the ten research articles included in this supplement issue. ICIBM 2016 included four workshops or tutorials, four keynote lectures, four conference invited talks, eight concurrent scientific sessions and a poster session for 53 accepted abstracts, covering current topics in bioinformatics, systems biology, intelligent computing, and biomedical informatics. Through our call for papers, a total of 77 original manuscripts were submitted to ICIBM 2016. After peer review, 11 articles were selected in this special issue, covering topics such as single cell RNA-seq analysis method, genome sequence and variation analysis, bioinformatics method for vaccine development, and cancer genomics.

## Introduction

The 2016 International Conference on Intelligent Biology and Medicine (ICIBM 2016) was held from December 8th to 10th, 2016 in Houston, Texas, USA. This is the fifth annual ICIBM conference. ICIBM conference series have two main aims: 1) to foster interdisciplinary and multidisciplinary research in bioinformatics, systems biology, intelligent computing, bioengineering, and data sciences, and 2) to provide an educational program for trainees and young investigators across a range of scientific disciplines to learn the frontier research in these areas and to build a network among both the established and junior investigators.

After 4 years of successful conference programs [1–4], ICIBM 2016 turned out to be the largest of all the ICIBM series in all aspects: number of attendees, talks, abstracts, posters, etc. It served as a platform for bringing together more than 150 scientists or trainees across the world with diverse backgrounds and training ranging from biology, medicine, computer science, bioengineering, bioinformatics, statistics, mathematics, and genomics, among others. We received 77 original manuscripts and 53 abstracts. These manuscripts and abstracts covered research topics including next-generation sequencing (NGS), genomics and other omics research, biological pathway and network analysis, computational algorithms, methods and tools. An emerging research area at ICIBM is data science especially in medical data. ICIBM 2016 brought a special session on this specific topic. Thanks to the grant support from the National Science Foundation, we were able to provide 22 travel awards to trainees from diverse backgrounds across both the USA and international institutions. As before, we formed the Award Committee to review and select the travel awards based on the quality of the research (paper or abstract), the financial need, and diversity/minority of the attendees. In the following section, we summarize the scientific program of the ICIBM 2016 and provide an editorial report of the 11 research articles included in this BMC Genomics supplement issue.

## ICIBM 2016 scientific program

The ICIBM 2016 spanned 3 days and its scientific program included 4 workshops/tutorials, 4 keynote lectures, 4 conference invited talks, 8 concurrent scientific sessions and a poster session with 53 accepted abstracts. The presentations covered emerging areas of bioinformatics, systems biology, intelligent computing, data sciences, and biomedical informatics. In the following sections, we briefly review the keynote lectures, conference invited talks, workshops/tutorials, scientific sessions and the poster session.

### Keynote lectures

Four world-renowned scientists presented keynote lectures on their innovative research and shared their perspectives. These speakers were Dr. Trey Ideker from the University of California at San Diego, Dr. John Weinstein from The University of Texas MD Anderson Cancer Center, Dr. Edward Marcotte from The University of Texas at Austin, and Dr. Yi Xing from The University of California at Los Angeles.

“*Siri of the Cell – Intelligent agents for systems medicine constructed using systems data*” by Dr. Trey Ideker. In the keynote lecture, Dr. Ideker introduced how we can feed omics data into multiscale models of the cell using similar intelligent agents, like Siri for cellular phone, to predict a range of cellular phenotypes and answer various biological questions. Dr. Ideker is a Professor of Genetics in the Department of Medicine at the University of California at San Diego (UCSD). He serves as the Director of the San Diego Center for Systems Biology. He is also the adjunct professor of Computer Science and Bioengineering and member of the Moores UCSD Cancer Center. Dr. Ideker has founded influential bioinformatic tools including Cytoscape, a popular network analysis platform which has been cited >12,000 times. He serves on the Editorial Boards for Cell, Cell Reports, Nature Scientific Data, EMBO Molecular Systems Biology, and PLoS Computational Biology and is a Fellow of AAAS and AIMBE.

*“Evolution and the proteome: Insights into protein function from deeply conserved gene modules”* by Dr. Edward Marcotte. Dr. Marcotte described his approaches to test the predictive information of the deeply homologous genes and pathways. He discussed his search for new models of human disease among phenotypes of distant organisms, his attempts to systematically humanize yeast cells, and his program to apply high-throughput protein mass spectrometry in order to measure conserved physical interactions among the thousands of proteins shared across the eukaryotic tree of life. Dr. Marcotte is a Professor in the Department of Molecular Biosciences at the University of Texas, where he holds the Mr. and Mrs. Corbin J. Robertson, Sr. Regents Chair in Molecular Biology and co-directs the UT Center for Systems and Synthetic Biology. His research falls in the general areas of proteomics, bioinformatics, and systems and synthetic biology, with current work focused on the interactions, dynamics, and evolution of proteins across the tree of life. He has received a National Institutes of Health Director’s Pioneer Award, and is a Fellow of the Royal Society of Chemistry, the American Academy of Microbiology, and the American Association for the Advancement of Science.

*“Molecular profiling of cancers: The Cancer Genome Atlas project and beyond”* by Dr. John Weinstein. In the lecture, Dr. Weinstein shared the recent exciting findings as well as the challenges of The Cancer Genome Atlas (TCGA) project. TCGA currently constitutes 33 tumor types and provides molecular, pathological, and clinical data that can be mined for generations. Specifically, he pointed out that the sophisticated bioinformatics and computational tools developed around this data will be very beneficial in the long run. Dr. Weinstein is the Professor and Chair of the Department of Bioinformatics and Computational Biology at the University of Texas MD Anderson Cancer Center. He has over more than 300 publications, including 15 as first author in Science or Nature, 11 that have been cited in the literature more than 600 times each, and more than 40 in journals with impact factors higher than 30. He is cited as “a pioneer of the post-genomic era in biomedical science.” His research group applies a mix of genomic, proteomic, metabolomics, systems biological, and bioinformatic tools to the search of new biomarkers, prevention strategies, and therapies for cancer. He is the leader of many large-scale cancer research projects such as TCGA, CCLE, and GDSC.

*“Elucidate transcriptome isoform complexity using massive RNA-seq data”* by Dr. Yi Xing. Dr. Xing presented the recent advances in high-throughput RNA sequencing (RNA-seq) technology, which have provided a powerful tool for transcriptome-wide measurements of mRNA isoform complexity at an unprecedented resolution. He discussed his recent efforts in developing computational and statistical methods for elucidating transcriptome isoform complexity using massive RNA-seq datasets. Dr. Xing is a Professor in the Department of Microbiology, Immunology and Molecular Genetics at University of California at Los Angeles (UCLA) and the director of UCLA’s Bioinformatics and Interdepartmental PhD Program. His research focuses on combining genomic, bioinformatics, molecular, and systems approaches to elucidate the variation and dynamics of RNA regulatory networks in development and disease. Dr. Xing has received prestigious young investigator research awards from the Sloan Foundation and the March of Dimes Foundation.

### Conference invited talks

ICIBM 2016 featured four conference invited talks covering frontier and emerging research topics such as novel regulatory roles of 3′ untranslated region (UTR) shortening in tumorigenesis, tumor-stroma crosstalk in tumor microenvironment for drug combinations, tumor phylogeny inference using single-cell DNA sequencing data, and functional proteomics in precision cancer medicine, etc. Each speaker has developed cutting-edge technologies or methods in bioinformatics and genomics fields.

“3′-UTR shortening represses tumor suppressors in trans by disrupting ceRNA crosstalk” by Dr. Wei Li, professor at Baylor College of Medicine. Dr. Li presented that shortened 3′-UTRs in breast cancers are strongly associated with repression of tumor suppressors enriched in competing endogenous RNAs (ceRNAs). He also introduced his model-based analysis of the trans effect of 3′-UTR shortening (MAT3UTR), which predicts many trans-targets of 3′-UTR shortening, including PTEN, a crucial tumor suppressor involved in ceRNA crosstalk. Through big data analysis followed by functional validation, they found that the major role of 3′-UTR shortening in tumorigenesis is to repress tumor suppressors in trans, rather than induce proto-oncogenes in cis.

“Modelling tumor-stroma crosstalk in tumor microenvironment for drug combinations and biomarker discovery in cancer” by Dr. Stephen Wong, Chairman and Professor of Department of Systems Medicine and Bioengineering at Houston Methodist Hospital and Cornell University. In his talk, Dr. Wong reviewed a body of the computational work in his lab that model tumor microenvironment in cancer and the discovery of drug combination and biomarkers in cancer. His solid data demonstrated that bioinformatics approaches are important for drug and biomarker discovery in cancer.

“Tumor phylogeny inference from single-cell DNA sequencing data” by Dr. Ken Chen, Associate Professor at the Department of Bioinformatics and Computational Biology and the Director of Bioinformatics of Khalifa Institute of Personalized Cancer Therapy, the University of Texas MD Anderson Cancer Center. Dr. Chen talked about how he overcame some of the computational challenges by developing a suite of new algorithms for single-cell DNA sequencing technology in cancer research, including Monovar, SiFit, and novoBreak.

“Functional proteomics as a major approach for precision cancer medicine” by Dr. Han Liang, Associate Professor at the Department of Bioinformatics and Computational Biology, the University of Texas MD Anderson Cancer Center. Dr. Liang presented his work for evaluating biomarkers and elucidating the mechanisms underlying the sensitivity and resistance to cancer therapy using reverse-phase protein arrays (RPPAs) data. His team developed protein-based prognostic models for stratifying patients into different risk groups by integrating RPPA data and TCGA data from independent patient cohorts. His work demonstrates the utility of RPPA as a powerful approach in prognostic and therapeutic strategies for precision cancer medicine.

### Workshops

ICIBM 2016 included four workshops/tutorials, which covered important techniques such as pathway and network analysis, statistical clustering analysis of multi-omics data, high throughput data analysis, and ENCODE data analysis and visualization. These workshops/tutorials were well attended and appreciated by the conference participants. They discussed the various strategies and at times provided the attendees with hands-on exercise using open source tools.

*“Tutorial: Introduction to pathway and network analysis.”* This tutorial was organized by Dr. Amin Momin from MD Anderson Cancer Center. Dr. Momin introduced the attendees about pathway and network analysis, which have mainly emerged as two robust technologies that provide biological annotation based on empirical and predicted findings. The participants also had an opportunity to do some hands-on exercise using open source tools to practice these techniques.

*“Tutorial: Statistical methods for integrative clustering analysis of multi-omics data.”* This tutorial was organized by Dr. Qianxing Mo from Baylor College of Medicine. Dr. Mo introduced the iClusterPlus method and a newly developed statistical method for integrative clustering analysis of the multi-omics data. He also introduced the theoretical background of the integrative clustering methods and illustrated the methods using TCGA multi-omics cancer data. Through the tutorial, attendees obtained hands-on experience with integrative clustering analysis of multi-omics data.

*“Workshop: High throughput data analysis, DNA, RNA, microbiome sequencing and genotyping.”* Organizer Dr. Yan Guo from Vanderbilt University Medical Center introduced the background, quality control, processing and interpretation for each of the four types of high throughput genomic data. This workshop helped attendees understand the technology background and the standard analysis pipeline and interpretation of the results for each type of genomic data.

*“Workshop: Interactive workshop on ENCODE data analysis and visualization.”* Organizer Dr. Yue Feng from Pennsylvania State University College of Medicine illustrated and provided hands-on tutorial on using ENCODE portal website, including how to browse, search and download ENCODE data. He introduced users to ENCODE analysis pipeline and REST APIs, explained annotations (such as enhancers and promoters) and its query website, and demonstrated how to visualize the 3D genome organization data (Hi-C and ChIA-PET) generated by ENCODE consortium.

### Scientific sessions

ICIBM 2016 had eight concurrent scientific sessions, which were incredibly valuable to research, education and innovation. Speakers were chosen from those top ranked manuscripts after peer review and they were from various fields of bioinformatics, genomics, systems biology, intelligent computing, and biomedical informatics. The eight session are:

Session I: Genome Structure Analysis and Visualization

Session II: NGS Analysis and Tools I

Session III: Computational Drug Discovery

Session IV: Medical Informatics and Big Data

Session V: NGS Analysis and Tools II

Session VI: Cancer Genomics

Session VII: Systems Biology

Session VIII: Big Data and Machine Learning

In addition, our poster session had a total of 53 abstracts for poster presentation. The topics included emerging research areas such as bioinformatics, genomics and genetics, next generation sequencing data analysis, big data science including storage, analysis, modelling and visualization, personalized medicine, drug discovery, drug designing, drug repurposing, proteomics, image analysis and processing, modelling and simulation of biological processes, pathways and networks, mathematical and quantitative models of cellular and multi-cellular systems, multi-dimensional omics data integration, metabolomics, application of systems biology approaches to biomedical sciences, machine learning, data mining, pattern recognition, natural language processing, literature mining, semantic oncology, neural computing, kernel methods, evolutionary computing, ensemble methods, manifold learning theory, artificial life and artificial immune systems.

The details of these sessions were included on the conference website as well as in the program book, which was handed out to the attendees during the conference. Here, we provide an editorial report of the supplements to BMC Genomics that include 11 research papers selected from 77 manuscripts submitted to ICIBM 2016. Each manuscript was reviewed by at least two reviewers (most by three reviewers) and was substantially revised by taking care of the reviewers’ critiques before further review and acceptance. Other selected papers were accepted in other BMC journals: BMC Systems Biology, BMC Bioinformatics, BMC Medical Informatics and Decision Making, and BMC Medical Genomics.

In the first paper, Yang et al. [5] introduced a novel algorithm, namely SAIC (Single cell Analysis via Iterative Clustering), that identifies the optimal set of signature genes from single cell RNA-seq data and separates single cells into distinct clusters. Their method utilizes an iterative clustering approach to perform an exhaustive search for the best parameters within the search space. The outcome is to identify signature gene set that gives the best separation of the cell clusters. The authors applied SAIC to one simulation dataset and two published single cell RNA-seq datasets and found it performed better than PCA method based on DB index score.

Chi-square statistics based on word pattern frequencies have long been proposed for molecular sequence comparison, but the optimal size of word patterns is not well studied. In comparison of two Markovian sequences, Bai et al. [6] showed theoretically and by applications to simulated and real data that the optimal word size equals the maximum order of the two sequences plus one. This critical information is applicable to the comparison of both long and next generation sequencing (NGS) short reads data. This study provides guidance on the choice of word size in alignment sequence comparison.

Hampton et al. [7] presented a breakpoint calling tool, SVachra (Structural Variation Assessment of CHRomosomal Aberrations), that identifies large insertions or deletions, inversions, inter- and intra-chromosomal translocations utilizing both inward and outward facing read types generated by mate pair sequencing. They demonstrated that SVachra exhibited the highest validation rate and reported the widest distribution of SV types and size ranges when compared to other SV callers.

Detecting the difference of single nucleotide variant (SNV) called from DNA and RNA samples of the same subject has been an interest of researchers. Through careful quality control and analyses of SNVs inferred from ten subjects with five distinct types of high throughput sequencing data, Guo et al. [8] found high consistency between DNA-DNA pairs and lower consistency in DNA-RNA or RNA-RNA pairs. Majority of the DNA-RNA difference were due to technical errors. Their findings suggested that SNV detection using RNA-seq data is subject to high false positive rates.

Position weight matrix (PWM) and sequence logo are the most widely used representations of transcription factor binding site in biological sequences. Although there are a few tools to generate sequence logos from PWM, there is no tool does the reverse. In Gao et al. [9], the authors proposed logo2PWM for reconstructing PWMs from sequence logo images. Evaluation results on over one thousand logos from different sources showed that the correlation between the reconstructed PWMs and the original PWMs were constantly high. logo2PWM may benefit the study of transcription by filling the gap between sequence logo and PWM.

Sher et al. [10] presented a new machine learning system which combines deep neural networks, analytical learning, and text mining techniques to predict epitopes using continuous primary protein sequence as input. They combined these concepts together to produce a pipeline called DRREP (Deep Ridge Regressed Epitope Predictor). DRREP was benchmarked against other state of the art epitope predictors, and achieved impressive improvements over other methods.

Apaydin et al. [11] study the validity and robustness of existing bi-level methods for strain optimization under uncertainty and non-cooperative environment. Specifically, the authors proposed new pessimistic optimization formulations: P-ROOM and P-OptKnock, aiming to derive robust mutants with the desired overproduction under two different existing mutant cell survival models, ROOM and OptKnock. Their pessimistic strain optimization methods could produce more robust solutions regardless of the inner-level mutant survival models, which are desired as the models for cell survival are often approximate to real-world systems.

The incidence of kidney renal clear cell carcinoma (KIRC) is expected to continue to increase in the US. There is an urgent need to find effective diagnostic biomarkers for KIRC that could help earlier detection and customized treatment strategies. Using the data from TCGA, Han et al. [12] found KIRC had many more protein prognostic biomarkers of survival time than other cancer types. They identified 52 genes as well as 4 tumor-stage-specific genes whose mRNA and protein expression were prognostic biomarkers of KIRC survival. The study indicated that these biomarkers might have clinical values for KIRC.

In another paper, Kuznetsov et al. [13] studied microRNA signatures in 582 high-grade serous ovarian carcinomas (HG-SOC). They revealed two robust and unbiased microRNA-based prognostic classifiers. They predicted specific target genes involved in nine cancer-related and two oocyte maturation pathways. Each gene in these pathways is regulated by more than one microRNA of the distinct microRNA-based prognostic classifiers. In total, the authors identified three HG-SOC subtypes and suggested possible microRNA-controlled common pathophysiological mechanisms.

In the next paper, the authors [14] developed a systematic workflow to screen for key modulators on a genome-wide scale based on gene expression profiles in cancer. They applied the method to a dataset of 286 breast tumors. They identified nearly a thousand key modulators, and verified the results in three independent cohorts. These modulators were involved in immune response and hormone signaling. The study provided candidates for further biological investigation in breast cancer.

In the last paper, Xu et al. [15] investigated the circular RNA (circRNAs) expression profiles and features in ten human tissues. Even though 33 circRNAs were found to be expressed ubiquitously in all adult tissues, hundreds of circRNAs have tissue specific expressions. Non-tissue specific circRNAs were further analyzed through a circRNA-miRNA-mRNA regulatory network. Furthermore, higher expression level of circRNAs in mammary gland than other tissues might be attributed to the rich innervation. Overall, this comprehensive study proposed that circRNAs play their roles in a tissue-specific and development-specific manner.

## Conference organization

### 2016 international conference on intelligent biology and medicine (ICIBM 2016)

(December 8–10, 2016, Houston, TX, USA)

We would like to express our sincere gratitude to the members of the Steering, Program, Publication, Workshop/Tutorial, Publicity, Award, Trainee and Local Organization Committees, as well as to all the reviewers and volunteers, who spent their valuable time and effort on making ICIBM 2016 a success. The conference accomplishments are the results of support and hard work of all these people.

#### Hosts and sponsors

The University of Texas Health Science Center at Houston (UTHealth), National Science Foundation, The University of Texas at San Antonio, Rice University, Baylor College of Medicine, Vanderbilt University, UTHealth Center for Precision Health, Rice University BioScience Research Collaborative.

#### General chairs

Zhongming Zhao (The University of Texas Health Science Center at Houston) and Jiajie Zhang (The University of Texas Health Science Center at Houston).

#### Steering committee

Wenjin J. Zheng (The University of Texas Health Science Center at Houston), Yidong Chen (The University of Texas Health Science Center at San Antonio), Tony Hu (Drexel University), Han Liang (MD Anderson Cancer Center), Steve Wong (Methodist Hospital/Cornell University), Michael Zhang (The University of Texas at Dallas).

#### Program committee

Chair: Zhandong Liu (Baylor College of Medicine), Co-Chair: Genevera Allen (Rice University). Members: Kristen Anton (Dartmouth College), Yongsheng Bai (Indiana State University), William S. Bush (Case Western Reserve University), Jake Chen (University of Alabama at Birmingham), Xue-Wen Chen (Wayne State University), Yidong Chen (The University of Texas Health Science Center), Lee Cooper (Emory University), Juan Cui (University of Nebraska, Lincoln), Qinghua Cui (Peking University, China), Youping Deng (Rush University Medical Center), Joshua Denny (Vanderbilt University), Jeremy Edwards (University of New Mexico Health Sciences Center), Weixing Feng (Harbin Engineering University, China), M Jennifer Fettweis (Virginia Commonwealth University), Marcelo Fiszman (National Library of Medicine, National Institutes of Health), Hsu Frank (Fordham University), Jan Freudenberg (Feinstein Medical Research Institute), Ge Gao (Peking University, China), Chittababu Guda, (University of Nebraska Medical Center), Yan Guo (Vanderbilt University), Steve Horvath (University of California, Los Angeles), Tzu-Hung Hsiao (The University of Texas Health Science Center at San Antonio), Kun Huang (The Ohio State University), Weichun Huang (National Institute of Environmental Health Sciences), Jenn-Kang Hwang (National Chiao Tung University, Taiwan), Peilin Jia (The University of Texas Health Science Center at Houston), Jim W. Zheng (The University of Texas Health Science Center at Houston), Judith Klein-Seetharaman (University of Warwick, UK), Jun Kong (Emory University), Dmitry Korkin (Worcester Polytechnic Institute), K.B. Kulasekera (University of Louisville), Fuhai Li (The Ohio State University), Leping Li (National Institute of Environmental Health Sciences), Liao Li (University of Delaware), Honghuang Lin (Boston University), Chunyu Liu (University of Illinois at Chicago), Hongfang Liu (Georgetown University), Qi Liu (Vanderbilt University), Tianming Liu (University of Georgia), Yin Liu (The University of Texas Health Science Center at Houston), Yunlong Liu (Indiana University), Xinghua Lu (University of Pittsburgh), Zhiyong Lu (NCBI, National Library of Medicine, NIH), Patricio A Manque (Universidad Mayor, Chile), Tabrez Mohammad (The University of Texas Health Science Center at San Antonio), Ranadip Pal, Texas Tech University), Yonghong Peng (University of Bradford, UK), Horacio Perez-Sanchez (Catholic University of Murcia, Spain), Jiang Qian (Johns Hopkins University), Thomas Rindflesch (National Institutes of Health), Marylyn Ritchie (Penn State University), Bairong Shen (Soochow University, China), Alexander Statnikov (New York University Langone Medical Center), Jingchun Sun (The University of Texas Health Science Center at Houston), Wing-Kin Sung (National University of Singapore, Singapore), Manabu Torii (Kaiser Permanente Southern California), Jun Wan (John Hopkins University), Edwin Wang (National Council of Canada/McGill University, Canada), Guohua Wang (Harbin Institute of Technology, China), Jing Wang (Baylor College of Medicine), Junbai Wang (Oslo University Hospital, Norway), Kai Wang (Columbia University), Qingguo Wang (Memorial Sloan Kettering Cancer Center), Xiaoyan Wang (University of Connecticut), Chaochun Wei (Shanghai Jiao Tong University, China), Xiwei Wu (City of Hope National Medical Center), Yonghui Wu (The University of Texas Health Science Center at Houston), Junfeng Xia (Anhui University, China), Lu Xie (Shanghai Center for Bioinformation Technology, China), Hua Xu (The University of Texas Health Science Center at Houston), Jianhua Xuan (Virginia Tech), Zhenqing Ye (The University of Texas Health Science Center at San Antonio), Sungroh Yoon (Seoul National University, Korea), Bing Zhang (Vanderbilt University), Michelle Zhang (The University of Texas at San Antonio), Yanqing Zhang (Georgia State University), Min Zhao (University of the Sunshine Coast, Australia), Huiru (Jane) Zheng (University of Ulster, UK), Dongxiao Zhu (Wayne State University).

#### Publication committee

Chair: Jianhua Ruan (The University of Texas at San Antonio), C-Chair: Yin Liu (The University of Texas Health Science Center at Houston).

#### Workshop/tutorial committee

Chair: Ken Chen (MD Anderson Cancer Center), Co-Chair: Yan Guo (Vanderbilt University).

#### Publicity committee

Chair: Degui Zhi (The University of Texas Health Science Center at Houston).

#### Award committee

Chair: Hua Xu (The University of Texas Health Science Center at Houston), Co-Chair: Yufei Huang (The University of Texas at San Antonio).

#### Trainee committee

Chair: Adriana Rubio (The University of Texas Health Science Center at Houston), Co-Chair: Andrew Laitman (Baylor College of Medicine).

#### Local organization committee

Chair: W. Jim Zheng, Co-Chair: Leng Han. Members: Smita Saraykar, Rosalinda Molina, Yukiko Bryson, Marcos Hernandez, Virginia M. Solt, Ryan Bien, all at The University of Texas Health Science Center at Houston.
